# Sponsorship Bias in Randomised Controlled Trials on Postoperative Pain After Third Molar Extraction: A Meta‐Research Study

**DOI:** 10.1002/ejp.70213

**Published:** 2026-01-30

**Authors:** Carlos Eduardo Dutra Rufato, Mayara Colpo Prado, Bernardo Antonio Agostini, Mateus Bertolini Fernandes dos Santos, Rafael Sarkis‐Onofre

**Affiliations:** ^1^ Graduate Program in Dentistry, ATITUS Educação Passo Fundo Rio Grande do Sul Brazil; ^2^ Department of Prosthodontics, College of Dentistry University of Iowa Iowa City Iowa USA

**Keywords:** dentistry, metaresearch, oral surgery, postoperative pain, randomised controlled trial, sponsorship bias, third molar

## Abstract

**Background:**

To evaluate whether sponsorship influences the reporting of positive results and the occurrence of selective outcome reporting (SOR) in randomised controlled trials (RCTs) investigating pharmacologic interventions for postoperative pain management following third molar extraction.

**Methods:**

This meta‐research included RCTs comparing at least one active drug with placebo, two active drugs, or combination thereof, and reporting outcomes related to pain reduction after third molar extraction. Searches were conducted in PubMed, Scopus and Web of Science without date restrictions and last search was performed on 2024 August. Study selection was performed in Rayyan QCRI, with two independent reviewers screening titles, abstracts and full texts. Data extraction was also conducted independently by two reviewers, collecting information on year of publication, trial design, number of groups, placebo comparisons, sample size (number of patients/teeth), follow‐up losses, statistical significance of results, protocol registration and funding disclosures. Selective outcome reporting was assessed by comparing registered protocols with published outcomes. Associations between sponsorship status and both SOR and positive result reporting were analysed using chi‐square test (*α* = 0.05).

**Results:**

A total of 430 RCTs were included. No association was found between sponsorship status and SOR (*p* = 0.861), nor between sponsorship status and the reporting of positive results (*p* = 0.241).

**Conclusions:**

In this sample, sponsorship was not associated with either selective outcome reporting or the likelihood of reporting positive results in RCTs on postoperative pain management after third molar extraction.

**Significance Statement:**

The absence of sponsorship bias in RCTs on third molar extraction suggests that industry‐funded and non‐sponsored studies provide comparably reliable evidence, supporting clinicians in making unbiased, evidence‐based decisions for postoperative pain management in third molar surgery.

## 1 Introduction

Third molar surgery is one of the most common procedures in dentistry. The surgical removal of these teeth is frequently associated with postoperative pain, edema and trismus (Bouloux et al. 2007). This pain, typically ranging from moderate to severe, is usually managed with analgesics and anti‐inflammatory drugs. Despite widespread use of these medications, there is no established consensus on the optimal therapeutic strategy for managing postoperative pain following third molar extraction. Various medications and strategies for managing this acute pain continue to be investigated in randomised controlled trials (RCTs) (Best et al. 2017; Daniels et al. 2018; Gay‐Escoda et al. 2019; Abdelraziq et al. 2023; Langford et al. 2024).

Dentistry is rapidly entering a new era of evidence‐based practice, in which prevention and treatment must be supported by proven effectiveness. As evidence‐based practice becomes more widespread, professionals are expected to rely on the best available evidence when making clinical decisions (ADA 2013). RCTs represent the gold standard for evaluating health interventions and are considered one of the most reliable sources of scientific evidence. In general, RCTs findings help shape clinical protocols and guide therapeutic choices for patients with similar conditions (Pihlstrom et al. 2012; Friedman et al. 2015).

RCTs are a crucial step in drug development. Based on promising preclinical results, new medicines undergo phase I to III RCTs to evaluate safety and efficacy before approval (Umscheid et al. 2011; Pihlstrom et al. 2012). However, this process is lengthy, costly, and has a low probability of success: of every 10,000 compounds discovered, 250 reach preclinical trials, five enter RCTs, and only one is marketed (Schmidt 2001). The estimated cost ranges from 161 million to 2 billion dollars and is likely even higher today (Sertkaya et al. 2016). Given these demands, many RCTs are conducted with substantial industry funding, and the pharmaceutical industry remains one of the main sponsors of health research (Flacco et al. 2015; Moses et al. 2015; Holman and Elliott 2018).

Concerns have been raised that industry sponsorship may bias clinical research, particularly RCTs. Sponsorship can influence the design, conduct, outcomes and reporting of studies, since unfavourable results may pose financial risks for the sponsor. Reported mechanisms of sponsorship bias include eligibility criteria, sample size, allocation and blinding, choice of comparators, outcome assessment, statistical analyses and even practices, such as selective outcome reporting (Lundh et al. 2017; Holman and Elliott 2018; Catalogue of Bias 2019). In dentistry, studies on sponsorship bias have shown inconsistent findings: while some found no significant differences between sponsored and non‐sponsored trials, others reported more favourable outcomes in industry‐funded research (Popelut et al. 2010; Schwendicke et al. 2016; Reda et al. 2017; Santos et al. 2019; Saltaji et al. 2021).

Therefore, given the importance of pain management after third molar extraction, and the fact that this clinical model is widely used to investigate interventions and new therapies for pain and inflammation in both medicine and dentistry, the potential effects of industry sponsorship on such studies should be carefully examined. Thus, the objective of this study is to evaluate the effect of sponsorship on the reporting of positive results and the presence of selective outcome reporting in RCTs investigating drugs for postoperative pain management in patients undergoing third molar extraction.

## 2 Materials and Methods

This is a meta‐research study. The study protocol and all related materials are available on the Open Science Framework (OSF) at https://osf.io/7fnmj/. The protocol was registered prospectively in October 2024, and study selection began only after registration.

### 2.1 Eligibility Criteria

We included RCTs based on the concept of Friedman et al. (2015), ‘prospective studies that present random allocation of participants to receive test or control interventions based on a clinical outcome specified before the start of the study’.

RCTs had to test at least one active drug against placebo (e.g., paracetamol 750 mg vs. placebo), two active drugs (e.g., paracetamol 750 mg vs. paracetamol 500 mg), or any combination of active drug and/or placebo in a multi‐arm design, and report outcomes on pain reduction after third molar extraction, regardless of tooth position. Studies were included regardless of their design (whether split‐mouth, parallel, etc.), level of methodological reporting, pain assessment method and sponsor. Trials that test non‐conventional medicines (medical products and practices that are not part of standard medical care; i.e., meditation, reiki), local anaesthetics, vitamins, plant extracts and anxiolytics were excluded. However, only studies in English, Portuguese and Spanish were considered because the research team did not have access to funding or paid translation tools at the time of the study.

### 2.2 Search

Three electronic databases were searched without time restrictions (PubMed, Scopus and Web of Science). The search strategy was developed using PubMed MeSH terms and adapted for the other databases (Table 1) and the last search was performed on 2024 August.

**TABLE 1:** Search strategy.

PubMed ((((‘Molar, Third’[Mesh] OR ‘Molar, Third’ OR ‘Molars, Third’ OR ‘Third Molar’ OR ‘Third Molars’ OR ‘Teeth, Wisdom’ OR ‘Wisdom Teeth’ OR ‘Tooth, Wisdom’ OR ‘Wisdom Tooth’) AND (‘Tooth Extraction’[Mesh] OR ‘Tooth Extraction’ OR ‘Extractions, Tooth’ OR ‘Extraction, Tooth’ OR ‘Tooth Extractions’)) AND (‘Pain, Postoperative’[Mesh] OR ‘Pain, Postoperative’ OR ‘Pain Measurement’[Mesh] OR ‘Pain Measurement’)) AND (‘Pharmaceutical Preparations’[Mesh] OR ‘Pharmaceutical Preparations’ OR ‘Drug’ OR ‘Drugs’ OR ‘Pharmaceuticals’ OR ‘Pharmaceutical Preparation’ OR ‘Pharmaceutical’ OR ‘Preparation, Pharmaceutical’ OR ‘Product, Pharmaceutical’)) AND (((clinical[Title/Abstract] AND trial[Title/Abstract]) OR clinical trials as topic[MeSH Terms] OR clinical trial[Publication Type] OR random*[Title/Abstract] OR random allocation[MeSH Terms] OR therapeutic use[MeSH Subheading]))
Scopus ‘Molar, Third’ OR ‘Molars, Third’ OR ‘Third Molar’ OR ‘Third Molars’ OR ‘Teeth, Wisdom’ OR ‘Wisdom Teeth’ OR ‘Tooth, Wisdom’ OR ‘Wisdom Tooth’ AND ‘Tooth Extraction’ OR ‘Extractions, Tooth’ OR ‘Extraction, Tooth’ OR ‘Tooth Extractions’ AND ‘Pain, Postoperative’ OR ‘Pain Measurement’ AND ‘Pharmaceutical Preparations’ OR ‘Drug’ OR ‘Drugs’ OR ‘Pharmaceuticals’ OR ‘Pharmaceutical Preparation’ OR ‘Pharmaceutical’ OR ‘Preparation, Pharmaceutical’ OR ‘Product, Pharmaceutical’ AND ‘Randomized Controlled Trials’ OR ‘Clinical Trials, Randomized’ OR ‘Trials, Randomized Clinical’ OR ‘Controlled Clinical Trials, Randomized’
Web of Science ((((ALL = (Molar, Third OR Molars, Third OR Third Molar OR Third Molars OR Teeth, Wisdom OR Wisdom Teeth OR Tooth, Wisdom OR Wisdom Tooth)) AND ALL = (Tooth Extraction OR Extractions, Tooth OR Extraction, Tooth OR Tooth Extractions)) AND ALL = (Pain, Postoperative OR Pain Measurement)) AND ALL = (Pharmaceutical Preparations OR Drug OR Drugs OR Pharmaceuticals OR Pharmaceutical Preparation OR Pharmaceutical OR Preparation, Pharmaceutical OR Product, Pharmaceutical)) AND ALL = (Randomized Controlled Trials OR Clinical Trials, Randomized OR Trials, Randomized Clinical OR Controlled Clinical Trials, Randomized)

### 2.3 Screening

Study selection was conducted using the Rayyan QCRI online review platform. Initially, duplicates were removed, and a pilot test was performed to assess agreement between the two reviewers involved in this phase (CEDR and MCP). For this purpose, references were randomly selected in Microsoft Excel. The two reviewers then independently screened the studies by first analysing titles and abstracts to determine eligibility. Each record was classified as ‘include’, ‘exclude’ or ‘unclear’. Articles classified as ‘include’ or ‘unclear’ were retrieved for full‐text review and further eligibility assessment by the same two reviewers. Discrepancies at both the title/abstract and full‐text screening stages were resolved through discussion, and if disagreement persisted, a third reviewer was consulted (RSO).

### 2.4 Data Collect

A standardised extraction form was developed in Microsoft Excel (see protocol). Initially, ten studies from the subset of final included studies were used to pilot test the form and ensure consistency in item interpretation. This pilot was conducted through discussion among the three reviewers involved in this phase to examine all extracted data. Subsequently, two reviewers (CEDR and MCP) independently extracted data from half of the included studies each, and a third reviewer verified consistency (RSO). Disagreements were resolved by consensus.

The following data were extracted: year of publication; RCT design (crossover, parallel, factorial or split‐mouth); number of study groups; comparison with placebo (yes/no); number of patients/teeth included; loss to follow‐up (yes/no/not reported); presence of statistically significant results for the outcome used to measure pain (no or yes); and reporting of an RCT registry or publicly available protocol (yes/yes, but no access/not reported), along with the timing of registration (prospective, retrospective or unclear). If the protocol was registered before the enrollment of the first participant, it was classified as prospective. Conversely, if registration occurred after participant enrollment had already begun, it was classified as retrospective. When insufficient information was available to determine the timing of registration, the classification was considered unclear.

Funding statements were collected from any point in the text and classified according to the study by Reda et al. (2017) into (a) sponsored—studies that clearly stated financial sponsorship from industry or declared any conflict of interest regarding sponsorship; (b) possibly sponsored—studies that reported receiving sponsorship, but where it was not possible to determine whether the funding source was for‐profit or non‐profit; (c) not sponsored—studies that clearly stated that they did not receive any financial support from industry, or that public funding was acquired; and (d) not reported—did not report any information about sponsorship. The sponsor of the study was also collected. If the authors report receiving a donation of any research material from a company or foundation, it was considered as sponsored.

Furthermore, studies that reported registering a protocol available were assessed for protocol deviation. The search for protocols was performed in the locations reported by the authors. The primary and secondary outcomes were collected from both the protocol and the article. If the study analysed only one outcome and did not specify it as the primary outcome, we considered as a primary. Also, we considered as a primary outcome, the outcome used in the sample size calculation. However, in cases where multiple outcomes were analysed without specifying which is primary and which is secondary, we considered as ‘unclear’. The occurrence of protocol deviation regarding selective reporting of results was analysed according to a checklist adapted from previous studies (Chan et al. 2004; Sendyk et al. 2021; Elagami et al. 2023): (a) primary outcome in the registry reported as secondary in the publication (primary outcome downgrade); (b) secondary outcome in the registry reported as primary in the publication (secondary outcome upgrade); (c) a new primary outcome (i.e., an outcome that was not described in the registry) introduced in the publication; (d) primary outcome in the registry omitted in the publication; (e) discrepancy in the primary outcome time frame (i.e., the timing of assessment of the primary outcome differed between the registry and the publication); (f) addition of a new secondary outcome in the final publication. In all such cases, we considered it a discrepancy between the protocol and the publication. When no discrepancies were identified, the study was classified as ‘no discrepancy’. When an outcome was reported in the protocol but not clearly addressed in the article, it was classified as ‘unclear’.

### 2.5 Data Analysis

Analyses were performed using Jamovi software, version 2.6 (available at: https://www.jamovi.org). Descriptive analyses were conducted according to the different categories of sponsorship. Quantitative variables are presented as median (minimum–maximum), and qualitative variables as frequencies and percentages. The chi‐square test was used to assess associations between sponsorship status and the two other variables: selective outcome reporting and the reporting of positive results, all categorised as mentioned before. A significance level of 5% was adopted for all analyses. As a sensitivity analysis, we conducted an assessment excluding trials classified as ‘not reported’ in the sponsor status.

### 2.6 Changes in Protocol

In the initial protocol, the objective was to evaluate the impact of sponsorship on pain effect measures. However, due to the substantial heterogeneity among the included studies, this analysis was not feasible. In addition, the presence of spin in these studies was initially planned for assessment but was not evaluated. Thus, the main change from the initial protocol was the evaluation of selective outcome reporting and the influence of sponsorship on this outcome. All extracted data, including those not analysed, are available on the Open Science Framework platform (see link below).

## 3 Results

**FIGURE 1:**
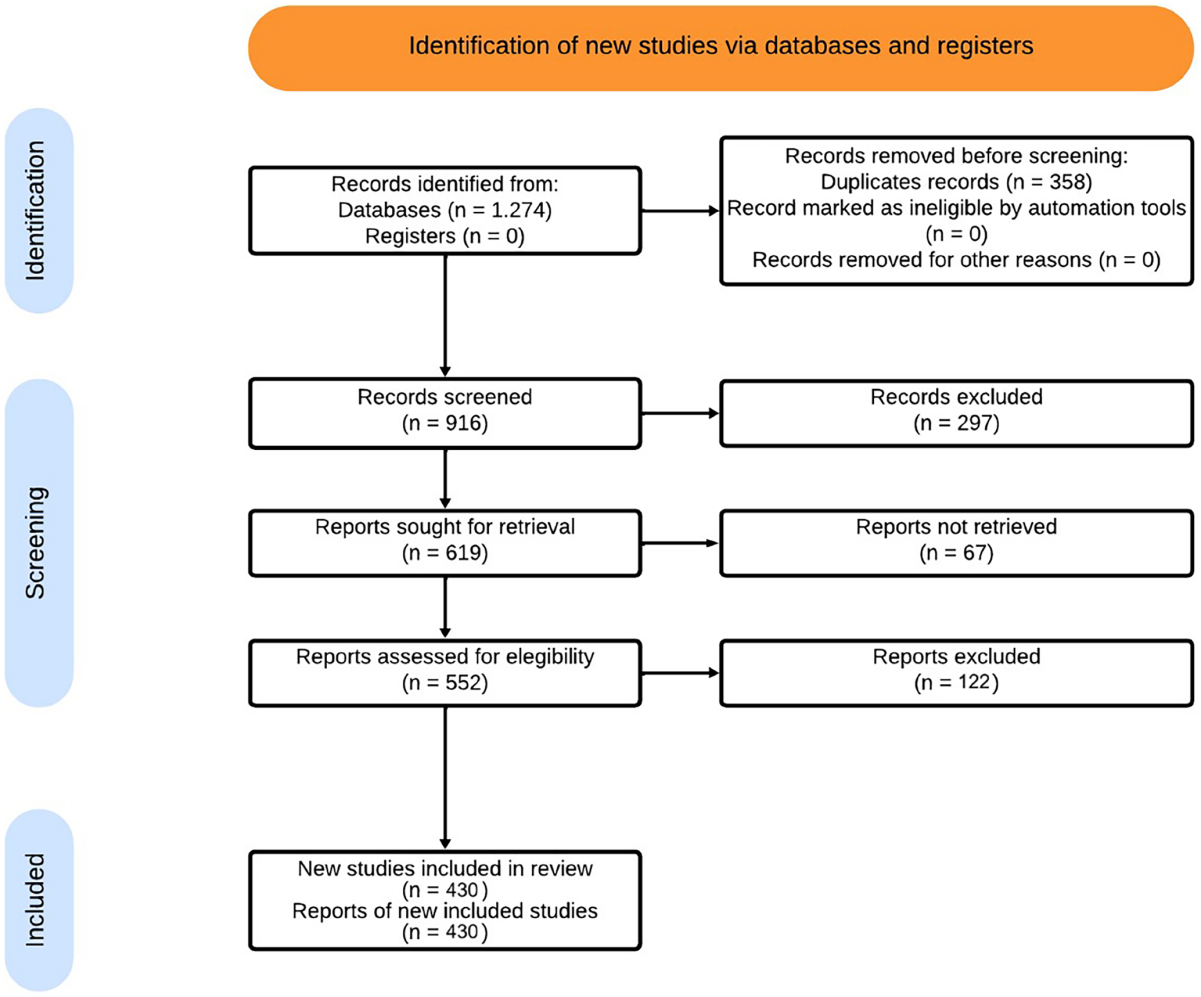
Flow diagram of the review process.

The search conducted in the selected databases identified 1274 studies, of which 358 were duplicates and removed, resulting in 916 unique articles. After screening titles and abstracts, 297 articles were excluded, leaving 619 studies for full‐text assessment. Full texts for 67 studies could not be retrieved, leaving 552 articles for detailed evaluation against the eligibility criteria. After excluding 122 studies, 430 RCTs were included in this meta‐research. Figure 1 illustrates the study selection process. The lists of included and excluded articles (with corresponding reasons), as well as the characteristics of each study and the data used in the analyses, can be accessed at the following link: https://osf.io/7fnmj/overview.

Table 2 summarises the characteristics of the included studies by sponsorship status. Considering individualised categories most part of the studies did not report details about sponsorship (*n* = 182; 42.3%), whereas 128 (29.8%) were classified as sponsored. The median number of patients was 82 (range: 10–997), and the median number of teeth was 80 (range: 20–1788). Sponsored studies had a higher median number of both patients (199) and teeth (188). Most trials employed a parallel design (*n* = 307; 71.4%) and included two comparison groups, whereas sponsored trials more often included three or more groups. The majority of studies compared interventions with placebo (*n* = 289; 67.2%) and reported loss to follow‐up (*n* = 220; 51.2%). Protocol registration was infrequent, with only 70 studies (16.3%) reporting protocol registration, and among these, most were registered prospectively (*n* = 49, 70%).

**TABLE 2:** Characteristics of the included studies according to sponsorship status.

	Total	Sponsored (*n* = 128)	Possibly sponsored (*n* = 22)	Unsponsored (*n* = 98)	Not reported (*n* = 182)
Number of patients (range)	82 (10–997)	199 (12–735)	103 (11–324)	60 (16–997)	67 (10–600)
Number of teeth (range)	80 (20–1788)	188 (30–1788)	98 (22–396)	73 (32–380)	72 (20–528)
Range of year of publication	1975–2024	1975–2024	1978–2023	1978–2024	1978–2024
Study design	**(*n*, %)**	**(*n*, %)**	**(*n*, %)**	**(*n*, %)**	**(*n*, %)**
Crossover	93 (21.6)	16 (17.2)	7 (7.5)	20 (21.5)	50 (53.8)
Factorial	1 (0.2)	0	0	0	1 (100)
Parallel	307 (71.4)	112 (36.5)	15 (4.9)	60 (19.5)	120 (39.1)
Split‐mouth	29 (6.7)	0	0	18 (62.1)	11 (37.9)
Number of study groups					
2	184 (42.8)	29 (15.8)	10 (5.4)	54 (29.3)	91 (49.5)
3	116 (27)	33 (28.4)	7 (6)	28 (24.1)	48 (41.4)
4	70 (16.3)	27 (38.6)	3 (4.3)	11 (15.7)	29 (41.2)
5 or more	60 (13.9)	39 (65)	2 (3.3)	5 (8.3)	14 (23.3)
Comparison with placebo					
Yes	289 (67.2)	113 (39.1)	15 (5.2)	57 (19.7)	104 (36)
No	141 (32.8)	15 (10.6)	7 (5)	41 (29.1)	78 (55.3)
Loss to follow‐up					
Yes	220 (51.2)	73 (33.2)	16 (7.3)	46 (21)	85 (38.6)
No	135 (17.4)	40 (29.6)	4 (2.9)	34 (25.2)	57 (42.2)
Not reported	75 (31.4)	15 (20)	2 (2.7)	18 (24)	40 (53.3)
Protocol registration					
Yes	70 (16.3)	24 (34.3)	3 (4.3)	31 (44.3)	12 (17.1)
Yes, but no access	6 (1.4)	0	1 (16.7)	3 (4.3)	2 (33.3)
Not reported	354 (82.3)	104 (29.4)	18 (5.1)	64 (18.1)	168 (47.5)
Timing of registration					
Prospective	49 (70)	19 (38.8)	2 (4.1)	17 (34.7)	11 (22.4)
Retrospective	17 (24.3)	2 (11.8)	1 (5.9)	13 (76.5)	1 (5.9)
Unclear	4 (5.7)	3 (75)	0	1 (25)	0

*Note:* Percentages in the *Total* column are calculated within the column. When considering sponsorship categories, percentages are calculated within each row for the respective factor.

From those with accessible protocols (*n* = 70) selective outcome reporting was identified in 38.6% of these studies, whereas 42.9% showed no discrepancies between protocol and publication. The most frequent discrepancy was the addition of a new secondary outcome in the final publication (*n* = 16; 59.3%). All the details analysed about selective outcome reporting could be found in Table 3.

**TABLE 3:** Frequency of selective reporting of outcome according to type of sponsor.

Presence of discrepancy	Total (*n* = 70)	Sponsored (*n* = 24)	Possibly sponsored (*n* = 3)	Unsponsored (*n* = 31)	Not reported (*n* = 12)
	(*n*, %)	(*n*, %)	(*n*, %)	(*n*, %)	(*n*, %)
Discrepancy	27 (38.6)	10 (37.0)	1 (3.7)	12 (44.4)	4 (14.8)
No discrepancy	30 (42.9)	11 (36.7)	1 (3.3)	11 (36.7)	7 (23.3)
Unclear	13 (18.6)	3 (21.1)	1 (7.7)	8 (61.5)	1 (7.7)

Types of discrepancy	Total (*n* = 27)	Sponsored (*n* = 10)	Possibly sponsored (*n* = 1)	Unsponsored (*n* = 12)	Not reported (*n* = 4)
	(*n*, %)	(*n*, %)	(*n*, %)	(*n*, %)	(*n*, %)
New secondary outcome was introduced in the publication	16 (59.3)	9 (56.3)	1 (6.3)	4 (25)	2 (12.5)
Primary outcomes in the registry were reported as secondary in the publication	3 (11.1)	0	0	3 (100)	0
Secondary outcomes in the registry were reported as primary in the publication.	4 (14.8)	1 (25)	0	2 (50)	1 (25)
The primary outcome in the registry was not reported in the paper	3 (11.1)	0	0	3 (100)	0
There was a difference between the registry and the publication regarding the timing of assessment of the primary outcome	1 (3.7)	0	0	0	1 (100)

*Note:* Percentages in the *Total* column are calculated within the column. When considering sponsorship categories, percentages are calculated within each row for the respective factor.

Table 4 presents the association between sponsorship status and two outcomes: selective outcome reporting and reporting of positive results. No significant association was found between sponsorship and selective outcome reporting (*p* = 0.861), nor between sponsorship status and the reporting of positive results (*p* = 0.241). Results of the sensitivity analysis are available on the Open Science Framework (OSF) at https://osf.io/7fnmj/.

**TABLE 4:** Associations between sponsorship status and selective outcome reporting and the reporting of positive results.

	Sponsored (*n*, %)	Possibly sponsored (*n*, %)	Unsponsored (*n*, %)	Not reported (*n*, %)	*p* ^b^
Selective outcome reporting					
Discrepancy	10 (37)	1 (3.7)	12 (44.4)	4 (14.8)	0.861
No discrepancy	11 (36.7)	1 (3.3)	11 (36.7)	7 (23.3)	
Statistically significant results^a^					
Yes	111 (31.6)	17 (4.8)	76 (21.7)	147 (41.9)	0.241
No	16 (21.1)	5 (6.6)	22 (28.9)	33 (43.4)	

^a^ A total of 427 studies were included in this analysis, as three studies did not perform statistical analysis and were therefore excluded.

^b^ *α* = 0.05.

## 4 Discussion

To the best of our knowledge, this is the first study to investigate the influence of sponsorship on RCTs investigating drugs for postoperative pain management in patients undergoing third molar extraction, specifically focusing on its impact on the reporting of positive results and the presence of selective outcome reporting. Our findings revealed no significant association between sponsorship status and these outcomes. This is noteworthy given that third molar extraction is one of the most frequently performed procedures in dentistry and serves as a widely accepted experimental model for testing analgesic drugs. Thus, our results do not provide evidence that industry sponsorship influenced the outcomes analysed, although limitations such as low protocol registration and the high proportion of ‘not reported’ sponsorship warrant cautious interpretation. The influence of industry sponsorship can occur at different stages of research, from its conception and design to the final reporting and dissemination of findings (Lundh et al. 2017; Holman and Elliott 2018; Catalogue of Bias 2019).

In the present study, we specifically evaluated this influence of industry sponsorship in terms of the reporting of positive results and selective outcome reporting. In dentistry, previous studies have examined the role of sponsorship across different outcomes and areas, but the heterogeneity of methodologies and endpoints makes direct comparison difficult and prevents firm conclusions (Popelut et al. 2010; Brignardello‐Petersen et al. 2013; Oomens et al. 2016; Schwendicke et al. 2016; Reda et al. 2017; Santos et al. 2019; Saltaji et al. 2021; Dini et al. 2022; Hu et al. 2022). This remains an area with considerable room for further investigation. When discussing the reporting of positive results, it is important to highlight the phenomenon of publication bias, which is not limited to sponsorship but reflects a broader tendency for journals to favour studies with statistically significant findings, regardless of sponsorship (DeVito and Goldacre 2019). However, in our study, we did not detect an association between sponsorship status and the reporting of positive results. This finding is particularly relevant, as it suggests that, in this field, industry does not appear to exert pressure toward the publication of favourable outcomes, thereby increasing the reliability of the available evidence.

Another important factor we evaluated was selective outcome reporting, a form of bias that occurs when a primary outcome of a clinical trial is modified or not published, or when a new outcome is introduced in the final report (Chan et al. 2004), generally discussed as outcome reporting bias. This issue can only be assessed when trial protocols are available, which highlights the importance of registering and publishing protocols for RCTs—a practice strongly encouraged by several initiatives and supported by dedicated platforms (Chan et al. 2025; Hopewell et al. 2025). Methodological studies have previously investigated selective outcome reporting in RCTs (Sendyk et al. 2019, 2021; Elagami et al. 2023; Santos et al. 2024; Fagoni et al. 2025). For example, Santos et al. (2024) evaluated selective outcome reporting in the same type of trials included in our study, although without assessing the role of sponsorship. Their prevalence estimates were very similar to ours. In addition, selective outcome reporting has also been reported in other fields of health research, with prevalence rates varying widely across studies (Sendyk et al. 2019, 2021; Elagami et al. 2023; Souza et al. 2023; Vitali et al. 2025). However, in our study no association was detected between sponsorship status and selective outcome reporting.

Only 70 studies included in our meta‐research had an accessible protocol, and among these, unsponsored trials showed the highest frequency of selective outcome reporting, followed by sponsored trials. The fact that protocols were available for such a small proportion of studies is itself an important negative finding and limits the external validity of selective outcome reporting estimates. This low rate of protocol registration may be partly explained by the inclusion of older trials, conducted before protocol registration became widely encouraged. Nonetheless, it also highlights a persistent gap in transparency and points to the need for improvements and broader adoption of protocol registration within dental research.

Another key observation was that a substantial proportion of trials failed to disclose sponsorship or funding information. This result is highly relevant, as disclosure of funding sources is a key practice for ensuring transparency and is strongly recommended by editorial policy committees as well as by reporting guidelines such as CONSORT and SPIRIT (Chan et al. 2025; Hopewell et al. 2025). The fact that many studies failed to provide this information suggests that the practice is still not widely adopted and highlights the need for stronger encouragement from journals and institutions. Moreover, the absence of sponsorship disclosure significantly affects both the transparency of RCTs and the interpretation of our findings, since it hinders a clear assessment of the actual impact of sponsorship on the evidence base. On the other hand, Prado et al. (2024) evaluated a sample of 844 randomised trials published between 2016 and 2021 and showed that most of them reported both conflict of interest and funding disclosure, indicating that these practices are becoming increasingly frequent in more recent publications. When considering the studies included in our analysis, published between 2004 and 2024, we observed that the majority did not have protocol registration, and a substantial proportion did not report funding disclosure.

Another important aspect highlighted in our study is the use of third molar extraction as an experimental model of postoperative pain to evaluate the analgesic efficacy of different drugs. This model is ethically justified by the clinical indication for third molar removal, since keeping the tooth in place may cause morbidity to the patient. It is well known that some degree of pain is expected after third molar extraction, and this pain is used as the target to be controlled or minimised with test drugs in RCTs (Reddy et al. 2012). Reddy et al. (2012) noted that experimental pain models in humans are essential for understanding pain mechanisms and appear to be ideal for testing analgesic compounds. Test drugs are not always developed specifically for dental pain, but they are often evaluated using this model. The reproducibility of the method is an important factor; if reproducibility is good, the model can be useful for drug screening, and randomised clinical trials are ideal for exploring the efficacy of clinical interventions.

This study has some limitations that should be acknowledged. At the methodological level, there was a language restriction; however, most relevant literature in this field is likely published in English, minimising potential bias. In addition, data extraction was not performed in duplicate, although a pilot test and the involvement of a third reviewer were implemented to ensure data consistency. Finally, publication bias was not empirically assessed, as this was outside the scope of the study. Nonetheless, it is important to note that 76 trials did not report statistically significant results, and the use of three major databases in our search strategy may have helped minimise the potential impact of publication bias. At the level of the included studies, there was considerable heterogeneity in outcomes and methodologies, which prevented a comparative analysis of pain reduction measures and limited our ability to assess the impact of sponsorship on effect sizes. Furthermore, a large proportion of trials did not report their sponsorship status, which requires cautious interpretation of the results.

Finally, it is important to emphasise that our analysis did not demonstrate an influence of sponsorship on the outcomes assessed. Although some studies on this topic have already been published in dentistry, there remains considerable room for progress, especially given the increasing number of RCTs in the field. As noted earlier, there is a tendency toward improvement in transparency practices, but further analyses across different areas of dentistry are still needed. In addition, efforts to standardise approaches for evaluating sponsorship bias would be valuable to strengthen the comparability and reliability of future evidence. Larger studies assessing the impact of sponsorship on the overall quality of trials are also important to determine whether industry‐funded studies differ from non‐sponsored ones, as this may have significant implications for the credibility and transparency of dental research and, consequently, in the process of decision‐making.

## 5 Conclusion

In conclusion, in the sample analysed, sponsorship was not associated with the reporting of positive results or with the presence of selective outcome reporting. We emphasise the importance of transparency in reporting all study details, including sponsorship status, to improve the reliability and credibility of clinical research.

## Author Contributions

Carlos Eduardo Dutra Rufato: project administration; methodology; supervision; validation; writing – review and editing. Mayara Colpo Prado: project administration; methodology; supervision; validation; writing – review and editing. Bernardo Antonio Agostini: project administration; methodology; supervision; validation; writing – review and editing. Mateus Bertolini Fernandes dos Santos: project administration; methodology; supervision; validation; writing – review and editing. Rafael Sarkis‐Onofre: project administration; methodology; supervision; validation; writing – review and editing.
